# Berberine-Mediated BCRP Inhibition Enhances Systemic Exposure of Rhein: A Study to Unravel the Pharmacokinetic Basis of Synergy in Da-Huang-Xiao-Shi Decoction

**DOI:** 10.3390/ph19030492

**Published:** 2026-03-17

**Authors:** Zhangyao Xu, Hongyu Li, Haoyu Xue, Xiaoge Wang, Tianming Wang, Yuyang Zhou, Jifeng Gu, Rong Shi

**Affiliations:** 1 Science and Technology Experimental Center, Shanghai University of Traditional Chinese Medicine, Shanghai 201203, China; xuyy0518@163.com (Z.X.); xhy2578832526@163.com (H.X.); wxg27@163.com (X.W.); yzhou78@uthsc.edu (Y.Z.); 2 Department of Pharmacy, Eye and ENT Hospital, Fudan University, Shanghai 200031, China; 3 Department of Pharmacology, School of Pharmacy, Shanghai University of Traditional Chinese Medicine, Shanghai 201203, China; wtmtcm@126.com; 4 Department of Pharmacology, Addiction Science, Toxicology, College of Medicine, University of Tennessee Health Science Center, Memphis, TN 38163, USA; 5 Shanghai Key Laboratory of Gene Editing and Cell Therapy for Rare Diseases, Shanghai 200031, China

**Keywords:** cholestasis, Da-Huang-Xiao-Shi Decoction, transporters, rhein, berberine

## Abstract

**Background/Objectives**: Cholestasis is a clinically intractable liver disorder. Da-Huang-Xiao-Shi Decoction (DHXSD), a classic traditional Chinese medicine formula, demonstrates notable efficacy, yet the mechanistic basis for its multi-herb synergy remains unclear. The purpose of this study was to decipher the pharmacokinetic interaction underlying the synergy of DHXSD. **Methods**: A cholestatic rat model was established in male Sprague Dawley rats. Hepatoprotective efficacy was evaluated, and the pharmacokinetics of anthraquinones were profiled. Key interaction mechanisms were investigated using the everted intestinal sac model, the breast cancer resistance protein (BCRP)-overexpressing MDCKII cells, and molecular docking simulations. **Results**: DHXSD provided significantly stronger hepatoprotection than its principal herb *Rheum palmatum* L. (DaHuang, DH) alone. This enhanced efficacy correlated with an approximate 2-fold increase in the systemic exposure of rhein compared to DH monotherapy. We identified berberine from *Phellodendron amurense* Rupr. (Huang Bo, HB) as the key synergist, which potently inhibited the BCRP efflux transporter, thereby enhancing rhein absorption. In contrast, geniposide from *Gardenia jasminoides* Ellis (Zhi Zi, ZZ) showed minimal effects. **Conclusions**: This work elucidates a concrete, transporter-mediated pharmacokinetic interaction as the core mechanism underlying herbal synergy in DHXSD. Our findings offer a rational strategy—targeted efflux transporter modulation—for improving the oral bioavailability of challenging drug molecules.

## 1. Introduction

Cholestasis, characterized by impaired bile flow and subsequent liver injury, remains a challenging hepatobiliary disorder with limited therapeutic options [1,2]. Traditional Chinese Medicine (TCM) formulas have emerged as promising candidates for cholestasis management, with several representative formulas demonstrating significant efficacy in both preclinical and clinical settings [3]. Da-Huang-Xiao-Shi Decoction (DHXSD), a classic TCM prescription recorded in Jin Gui Yao Lue, has been used for millennia to treat jaundice [4]. Modern studies have validated its potent anti-cholestatic effects against cholestasis in animal models induced by α-naphthylisothiocyanate (ANIT) [5,6] and in clinical trials.

DHXSD is composed of four medicinal herbs: *Rheum palmatum* L. (Da Huang, DH) and Mirabilitum (Purified Sodium Sulfate Decahydrate, Na_2_SO_4_·10H_2_O, MX) combined with *Phellodendron amurense* Rupr. (Huang Bo, HB) and *Gardenia jasminoides* Ellis (Zhi Zi, ZZ). While TCM theory attributes the formula’s efficacy to the synergistic interaction among these herbs, with DH and MX serving as the “principal” herbs and HB and ZZ as the “adjuvant” herbs to enhance effectiveness [7], an in vitro study has further suggested such interaction by showing that the complete formula markedly enhances the dissolution of rhein (a key constituent of DH) compared to DH alone [8]. This finding prompts a key question: could corresponding pharmacokinetic drug–drug interactions (PK-DDIs) occur in vivo, leading to increased systemic exposure of active constituents and underpinning the formula’s synergy?

From a pharmacokinetic perspective, co-administered substances can critically modulate the absorption, distribution, metabolism, and excretion of drugs [9,10]. A prime cellular determinant of oral drug absorption is the activity of efflux transporters, such as the Breast Cancer Resistance Protein (BCRP/ABCG2). BCRP, a pivotal member of the ATP-binding cassette superfamily, is highly expressed in the intestinal epithelium and actively extrudes a wide spectrum of xenobiotics and drugs back into the gut lumen, thereby limiting their oral bioavailability. Consequently, inhibition of intestinal BCRP is a well-established mechanism for clinically significant PK-DDIs that enhance drug exposure [11,12]. Notably, beyond synthetic drugs, numerous natural products and phytochemicals are potent modulators of BCRP, as evidenced by studies on compounds such as the flavonoids apigenin and quercetin, and the alkaloid berberine [13].

However, while interactions between single herbal constituents and transporters such as BCRP have been explored, the specific role of such transporters in mediating the complex herb–herb interactions within a complete classical formula remains largely unexplored. We therefore hypothesized that within DHXSD, berberine (from HB) enhances the intestinal absorption and systemic exposure of the active compounds by inhibiting BCRP-mediated efflux, thereby establishing a concrete pharmacokinetic mechanism that contributes to the formula’s overall synergy.

To test this hypothesis, this study was designed to first identify and quantify the in vivo PK-DDIs within DHXSD by comparatively profiling the systemic exposure of key anthraquinones from DH following administration of DH alone, the DH+MX pair, and the complete DHXSD formula in a cholestatic rat model. Subsequently, for the most significant interaction identified, we employed targeted in vitro and ex vivo models (everted gut sacs, cellular transport, and molecular docking) to mechanistically dissect the role of BCRP inhibition. This work aims to elucidate a pharmacokinetic basis for DHXSD’s herbal synergy and to establish a translatable strategy for mechanistically understanding complex herb–herb interactions in multi-component natural formulations.

## 2. Results

### 2.1. Effects of DHXSD and DH on Cholestatic Rats

**Figure 1:**
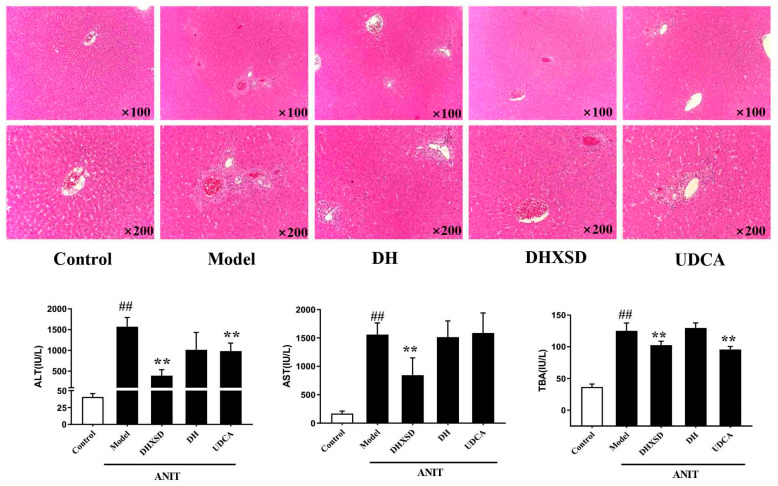
Effects of DHXSD and DH on cholestatic rats. Original magnification ×100 or ×200. Data are expressed as mean ± SD (*n* = 8). ^##^ *p <* 0.01 compared with the control group; ** *p <* 0.01 compared with the model group.

Compared to the control group, the model group exhibited a more than 5-fold increase in alanine aminotransferase (ALT) and aspartate aminotransferase (AST) levels and an approximately 3-fold rise in total bile acid (TBA) levels, indicating the occurrence of cholestasis. In contrast to the model group, the DH group did not significantly affect liver function in the modeled animals. However, DHXSD reduced ALT, AST, and TBA levels in the modeled animals (*p* < 0.05 or *p* < 0.01). Additionally, ursodeoxycholic acid (UDCA) significantly lowered ALT and TBA levels (*p* < 0.05 or *p* < 0.01) (Figure 1).

The model group exhibited characteristic histopathological features of acute cholestatic injury, including disrupted hepatic lobular architecture, central venous congestion, disorganized hepatic cords, hepatocyte swelling with vacuolar degeneration, and portal inflammation. In contrast to the DH group, which showed no significant improvement, both the DHXSD- and UDCA-treated groups demonstrated markedly reduced hepatic inflammation, ameliorated bile duct damage, and better-preserved hepatic microstructure (Figure 1).

### 2.2. Pharmacokinetic Profiles of Anthraquinones in Plasma of Cholestatic Rats After DH, DH+MX, and DHXSD Administration

**Figure 2:**
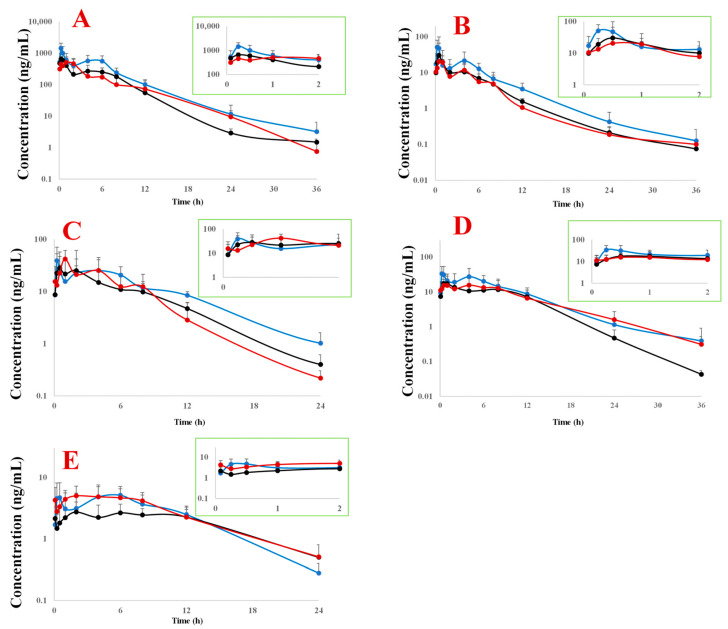
Plasma concentration–time profiles of anthraquinone components derived from Da Huang (DH), Da Huang plus Mang Xiao (DH+MX), and Da-Huang-Xiao-Shi Decoction (DHXSD) in cholestatic rats (mean ± SD, *n* = 6). The black, red, and blue curves represent the DH, DH+MX, and DHXSD groups, respectively. (**A**) Rhein; (**B**) Aloe emodin; (**C**) Emodin; (**D**) Chrysophanol; (**E**) Physcion.

In cholestatic rats administered DH, DH+MX, or DHXSD, five anthraquinone components were quantitatively detected in vivo: rhein, aloe emodin, emodin, chrysophanol, and physcion, with rhein exhibiting the highest plasma concentration and physcion the lowest. Compared to administration of DH, the DHXSD group showed a marked elevation in the maximum concentration (C_max_) of rhein, aloe emodin, and chrysophanol (*p* < 0.01), as well as physcion (*p* < 0.05). However, no such C_max_ enhancement was observed in the DH+MX group compared to the DH group. In addition to the C_max_ increase, the area under the curve (AUC) of rhein and aloe emodin was also markedly elevated in the DHXSD group. This increase was significant compared to the DH group (rhein, *p* < 0.01; aloe emodin, *p* < 0.05) and compared to the DH+MX group (rhein, *p* < 0.01; aloe emodin, *p* < 0.05) (Figure 2 and Table 1). Among these components, both the C_max_ and AUC of rhein exhibited an approximately 1.9-fold increase compared to DH alone, making it the most significantly affected component.

### 2.3. Effects of Berberine and Geniposide on Intestinal Flora Metabolism of Conjugated Rhein

**Figure 3:**
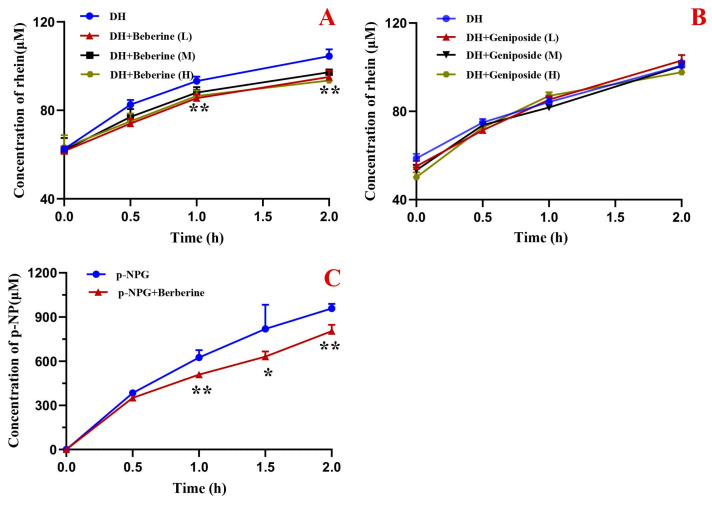
Metabolic fate of conjugated rhein in intestinal flora of cholestatic rats (mean ± SD, *n* = 3). (**A**) Effect of berberine on the conversion of conjugated rhein to free rhein. (**B**) Effect of geniposide on the conversion of conjugated rhein to free rhein. (**C**) Inhibitory effect of berberine on β-glucosidase activity using p-NPG as a substrate. Berberine and geniposide were tested at low (L, 100 μg/mL), medium (M, 300 μg/mL), and high (H, 1000 μg/mL) concentrations. * *p* < 0.05, ** *p* < 0.01 compared to the DH group (for (**A**,**B**)) or the p-NPG control group (for (**C**)).

As shown in Figure 3A, rhein levels increased with incubation time when DH extract was co-cultured with intestinal flora. After 2 h of incubation, the concentration rose by 67% (1.67-fold) compared to baseline, confirming the microbiota-mediated conversion of conjugated to free rhein. Compared to DH extract alone, co-treatment with berberine (100–1000 μg/mL) significantly suppressed rhein production at 1 and 2 h (*p* < 0.01), indicating inhibition of microbial deglycosylation. Notably, geniposide had no significant effect (Figure 3B), suggesting it does not modulate this metabolic pathway.

Further experiments revealed that the addition of berberine to bacterial cultures containing p-nitrophenyl-β-D-glucopyranoside (p-NPG) significantly decreased the generation of p-nitrophenol (p-NP) (*p* < 0.05 or *p* < 0.01, Figure 3C). Correlation analysis demonstrated a strong positive relationship between the concentrations of rhein and p-NP in the berberine-treated groups (r = 0.9904, *p* < 0.0001), indicating that berberine’s inhibitory effect on the metabolic activation of rhein is mediated through the inhibition of β-glucosidase.

### 2.4. Effect of Berberine and Geniposide on the Intestinal Transmembrane Transport of Rhein

**Figure 4:**
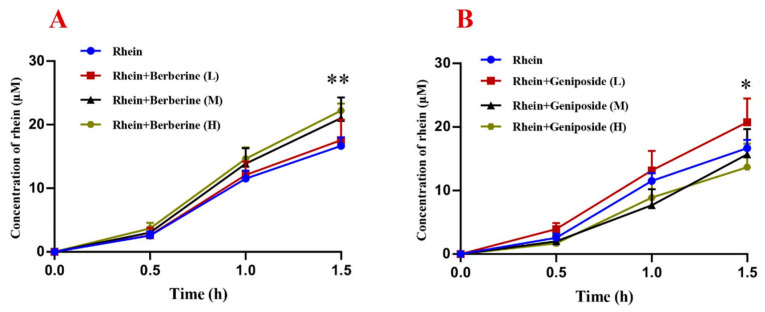
Effect of berberine and geniposide on the intestinal transmembrane transport of rhein (mean ± SD, *n* = 3). (**A**): Effect of berberine on rhein; (**B**): Effect of geniposide on rhein. Compounds were tested at low (L, 100 μg/mL), medium (M, 300 μg/mL), and high (H, 1000 μg/mL) concentrations. * *p* < 0.05, ** *p* < 0.01 compared to the “rhein alone” control.

Berberine at high concentrations (300 and 1000 μg/mL, M and H) significantly enhanced rhein’s intestinal transmembrane transport, while the low concentration (100 μg/mL, L) showed no effect (Figure 4A). As shown in Figure 4B, different concentrations of geniposide have different effects on the intestinal transmembrane transport of rhein. The addition of geniposide (100 μg/mL, L) promoted the intestinal absorption of rhein (*p* < 0.05). In contrast, geniposide (300 and 1000 μg/mL, M and H) had no significant impact.

### 2.5. Effect of Berberine and Geniposide on BCRP-Mediated Rhein Transport

**Figure 5:**
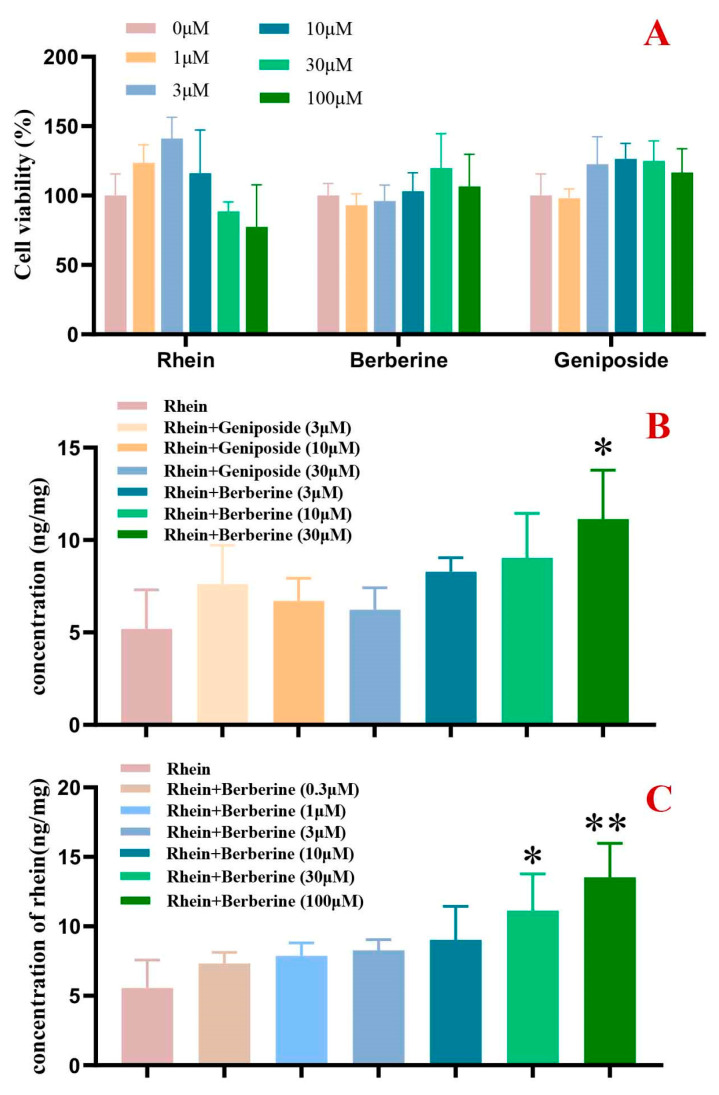
Effect of berberine and geniposide on BCRP-mediated rhein transport (mean ± SD, *n* = 3). (**A**): Effects of geniposide, berberine, and rhein (0–100 μM) on BCRP-MDCKII cell viability; (**B**): Effect of berberine and geniposide on BCRP-mediated rhein transport; (**C**): Concentration-dependent inhibitory effect of berberine on rhein. Compared to rhein alone, * *p* < 0.05, ** *p* < 0.01.

Results demonstrated that neither geniposide nor berberine (≤100 μM) significantly affected cell survival rates, while rhein showed no cytotoxicity at concentrations ≤ 30 μM (Figure 5A). Berberine (30 μM) markedly increased intracellular rhein accumulation (*p* < 0.05), suggesting the potent inhibition of BCRP-mediated rhein efflux (Figure 5B). In contrast, geniposide (3–30 μM) exhibited no effect. Dose–response analysis revealed that berberine (30–100 μM) enhanced intracellular rhein in a concentration-dependent manner, with statistically significant effects at 30 μM and 100 μM (Figure 5C), confirming its role as a BCRP transport inhibitor.

### 2.6. Binding Mode Analysis of Berberine at BCRP’s Substrate Pocket

**Figure 6:**
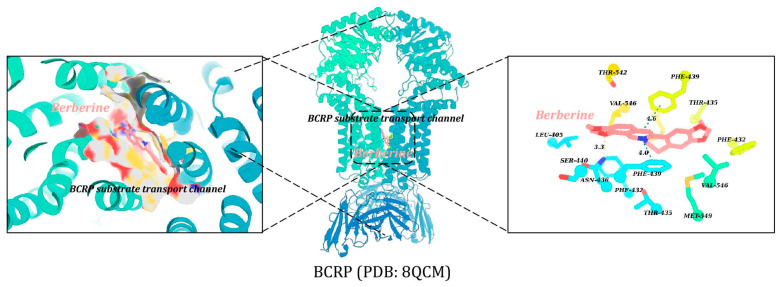
Binding mode analysis of berberine at the substrate pocket of BCRP.

Molecular docking analysis uncovered the likely inhibitory mechanism of berberine on BCRP (Figure 6). Berberine was found to bind tightly to BCRP’s substrate transport channel, forming a hydrogen bond with Asn436 (length 3.3 Å) and engaging in π-π stacking interactions with ChanA_Phe439 (distance 4.0 Å) and ChanB_Phe439 (distance 4.6 Å), with a docking score of −8.6 kcal/mol. This suggests that berberine may impede the transport of BCRP substrates such as rhein, by blocking the channel, thereby affecting BCRP’s function and reducing the efficiency of substrate efflux.

## 3. Discussion

As a cornerstone herb for cholestasis management in TCM [3,14,15], the therapeutic, particularly hepatoprotective, effects of DH in DHXSD originate from the synergistic contributions of its key anthraquinone constituents. Among them, rhein serves as a pivotal multi-target hepatoprotective agent with demonstrated anti-inflammatory, anti-fibrotic, and choleretic properties [16,17]. Concurrently, other major components, including emodin, aloe emodin, chrysophanol, and physcion (along with its glycosides), contribute to this network through their well-documented mechanisms of action, such as anti-inflammatory, antioxidant activities, and the regulation of cell death pathways [18,19,20,21]. Together, they form the material basis and multi-pathway network underlying the decoction’s hepatoprotective efficacy.

To evaluate the in vivo relevance of this compositional synergy, the clinical equivalent dose of DHXSD was selected based on its established efficacy in ANIT-induced cholestasis models. Consistent with the concept of herb–herb synergy, DH monotherapy (1.2 g/kg) failed to ameliorate ANIT-induced liver injury, whereas the complete formula (DHXSD) significantly improved serum ALT and AST levels, confirming that the therapeutic effect is combination-dependent. UDCA, used as a positive control, significantly reduced serum TBA and ALT levels, though its effect on AST was not statistically significant—a variability consistent with prior reports in cholestatic models [6,22]. This observed herb–herb interaction suggests potential underlying mechanisms at the pharmacokinetic level, which may enhance the systemic exposure and/or tissue distribution of active constituents. Therefore, to decipher the mechanism of this combination-dependent efficacy, we next focused on characterizing the PK-DDIs among the herbal components.

Pharmacokinetic profiling identified rhein as the dominant systemic anthraquinone post DHXSD administration, exhibiting 1.85-fold higher C_max_ and 1.96-fold greater AUC versus DH alone, despite a comparable elimination half-life. The observed variability in these parameters is likely attributable to the inherent biological heterogeneity of the cholestatic model, where individual differences in disease state and intestinal microbiota activity (critical for the hydrolysis of conjugated anthraquinones) can influence drug absorption. Importantly, although previous studies have reported that MX reduces anthraquinone bioavailability in normal animals [23], the DH+MX combination did not increase rhein exposure in our cholestatic model. The combination of elevated plasma exposure and an unchanged elimination phase, together with the lack of effect from MX, strongly suggests that the companion herbs (HB/ZZ) primarily enhance the intestinal absorption of rhein. Given that berberine (from HB) and geniposide (from ZZ) are major components of DHXSD [8], they were selected as the focus for subsequent mechanistic analysis.

Following oral administration, DHXSD components directly engage with intestinal microbiota—a potential hub for herb–herb interactions. This is particularly relevant for anthraquinones, which predominantly exist as water-soluble glycosides (conjugated forms) in the crude herb. These conjugates are poorly absorbed and must first be hydrolyzed by microbial β-glucosidases in the colon to release the active free aglycones (e.g., free rhein) for efficient systemic absorption [24]. Therefore, the intestinal microbiota plays a pivotal role in the biotransformation and bioavailability of anthraquinones. Existing evidence indicates that microbiota-derived β-glucosidases convert rhein–glucoside conjugates into absorbable free rhein [25,26]. Paradoxically, berberine (but not geniposide) inhibited this conversion in cholestatic model fecal samples—a phenomenon discordant with DHXSD’s in vivo rhein exposure enhancement. This apparent contradiction implies berberine’s β-glucosidase inhibition may be overshadowed by other factors in vivo.

Transmembrane transport emerged as another critical node of interaction. In the everted intestinal sac model, where tissue integrity was confirmed by stable lactate dehydrogenase (LDH) levels, berberine (300–1000 μg/mL) concentration-dependently enhanced rhein transport. In contrast, geniposide showed only a limited, dose-dependent effect, with a marginal enhancement observed at a low concentration (100 μg/mL) but no significant effect at its higher concentrations (300 and 1000 μg/mL). This, combined with its inactivity in the BCRP inhibition assay, indicates that the potential influence of geniposide on rhein is not mediated through BCRP inhibition. These findings indicate that berberine-mediated enhancement of rhein transmembrane transport partially underlies the increased systemic rhein exposure in DHXSD-treated cholestatic models.

Previous studies indicate that rhein undergoes passive diffusion into enterocytes but faces efflux transport mediated primarily by BCRP, with minimal involvement of P-glycoprotein proteins [27]. To investigate berberine’s absorption-enhancing effects, we employed BCRP-overexpressing MDCKII cells—a validated model for BCRP substrate/inhibitor screening [28]. Results demonstrate that berberine significantly inhibited BCRP-mediated rhein efflux, whereas geniposide was inactive. Consistent with cellular assays, molecular docking validated berberine as a BCRP inhibitor that impairs rhein efflux. Taken together, the data from the everted sac, cellular, and molecular docking studies provide a coherent mechanistic explanation for the apparent paradox. While berberine can inhibit microbial β-glucosidase activity in a controlled in vitro setting, its potent inhibition of the intestinal efflux transporter BCRP emerges as the dominant in vivo mechanism enhancing rhein absorption. This direct and robust effect on transmembrane transport at the enterocyte level overshadows the concurrent, partial suppression of microbial activation in the complex gut environment, thereby driving the net increase in rhein absorption and systemic exposure following DHXSD administration.

It should be noted that the present study focused on intestinal absorption mechanisms and did not investigate the potential role of hepatic transporters (e.g., BCRP) in the biliary excretion of rhein. Future studies measuring bile concentration or biliary clearance are warranted to fully elucidate the hepatobiliary disposition of rhein.

## 4. Materials and Methods

### 4.1. Materials

#### 4.1.1. Chemicals and Reagents

All crude drugs, including DH (Gansu, China), MX (Jiangsu, China), ZZ (Jiangxi, China), and HB (Sichuan, China), were supplied by Kangqiao Chinese Medicine Tablet Co., Ltd. (Shanghai, China), and authenticated according to the Chinese Pharmacopoeia (2020 edition). ANIT (purity ≥ 98%) was purchased from Sigma-Aldrich (St. Louis, MO, USA), and UDCA (batch L20094A) was obtained from Techpool Pharmaceutical Co., Ltd. (Shenzhen, China). The LDH Assay Kit was purchased from Nanjing Jiancheng Bioengineering Institute (Nanjing, China).

The reference standards, including rhein, emodin, chrysophanol, physcion, and geniposide were sourced from Yuanye Bio-Technology (Shanghai, China). Berberine and aloe emodin were procured from the National Institute for Pharmaceutical and Biological Product Control (Beijing, China). For use as internal standards (IS), mycophenolic acid (IS1) was acquired from Sigma-Aldrich (Buchs, Switzerland) and carbamazepine (IS2) was purchased from Sinopharm group (Shanghai, China). These reference materials possessed purity levels exceeding 98%. Additional High Performance Liquid Chromatography (HPLC)-grade reagents (acetic acid from Fisher Scientific, Waltham, MA, USA; ammonium acetate from CNW Technologies, Düsseldorf, Germany) and analytical-grade chemicals were used. The BCRP-MDCKII cell line was generously provided by Dr. Xiaodong Liu (Center of Drug Metabolism and Pharmacokinetics, China Pharmaceutical University, Nanjing, China).

#### 4.1.2. Animals

Experiments used male Sprague Dawley rats weighing between 230 and 250 g supplied by Vital River Laboratory Animal Technology Co., Ltd. (Beijing, China) (Certificate No. SCXK (Zhe) 2019-0001) and housed in the Animal Experiment Center of Shanghai University of Traditional Chinese Medicine. Animals were maintained under controlled conditions (room temperature: 22–25 °C; 12 h light/dark cycle) allowing unrestricted water intake. Prior to experiments, all animals underwent 12 h fasting while maintaining access to water. All experimental procedures met all ethical requirements for animal research at Shanghai University of TCM (Ethics Approval No. PZSHUTCM211129014). Animals were acclimatized to the housing conditions for at least one week before experiments. Healthy male rats within the specified weight range were included. No animals or data points were excluded from the analysis. Sample size was determined based on common practice for similar animal models.

### 4.2. Methods

#### 4.2.1. Sample Preparation

DHXSD was prepared as previously described [29]. Briefly, the botanical mixture (DH 12 g, HB 12 g, ZZ 15 grains) underwent dual aqueous extraction (1:10, *w*/*v*, 30 min per cycle). The combined filtrates were stirred with MX (12 g) until dissolution, then concentrated and then vacuum-dried at 60 °C. Extracts of DH alone and the DH+MX herb pair were also prepared, following the same extraction and drying protocol but with HB and ZZ omitted. For the DH+MX extract, MX (12 g) was added post extraction to the filtrates (consistent with the DHXSD method). The quantitative determination of the major constituents in the prepared decoctions (DH, DH+MX, and DHXSD) was conducted using a high-performance liquid chromatography–linear ion trap quadrupole–Orbitrap mass spectrometry method. A summary of the contents of these main compounds is provided in the Supplementary Materials.

#### 4.2.2. Experimental Design for Evaluating DHXSD and DH in Cholestatic Rats

(1) Animal dosing

Following acclimation, a random assignment procedure was used to divide the rats into five groups (*n* = 8 per group): control (normal saline), model (ANIT + saline), DH (ANIT + 1.2 g/kg DH, equivalent to the DH content in DHXSD), DHXSD (ANIT + 4.9 g/kg DHXSD, clinically equivalent dose as established in previous studies), and UDCA (ANIT + 60 mg/kg UDCA as positive control). All groups received daily oral gavage for 7 days. On day 5, ANIT (60 mg/kg in olive oil) [30] was administered to all groups except controls to induce cholestasis. Forty-eight hours post ANIT (day 7), blood and liver tissues were collected under anesthesia for analysis.

(2) Serum biochemistry and tissue examinations

Hepatic function was evaluated through measurement of serum ALT and AST activities, along with TBA concentrations using an automated analyzer (Hitachi 7080, Tokyo, Japan). Personnel performing the biochemical assays and histopathological evaluations were blinded to the group assignments of the samples. Liver samples were formalin-fixed, paraffin-embedded, and cut into 5 μm sections, and H&E-stained for histopathological evaluation (Nikon Eclipse Ci-L, Tokyo, Japan). The remaining serum and liver tissue samples were stored at −80 °C for subsequent analysis.

#### 4.2.3. Pharmacokinetic Study of Rhubarb Anthraquinones After Administration in Cholestatic Rats

Cholestasis was established in rats through a single oral dose of ANIT (60 mg/kg in olive oil). Random assignment procedure was used to divide the rats into three groups (*n* = 6 per group), receiving either: (1) DH (1.2 g/kg), (2) DH+MX (2.4 g/kg) or (3) DHXSD (4.9 g/kg) via oral gavage. Sequential blood samples were obtained at 5, 15, 30 min, and 1, 2, 4, 6, 8, 12, 24, and 36 h after administration. Plasma was separated by centrifugation and deproteinized by adding a 3:1 (*v*/*v*) ratio of acetonitrile containing 5 ng/mL IS1. Ultra-performance liquid chromatography–tandem mass spectrometry analysis was performed on the supernatants following a centrifugation, employing the described analytical approach [29]. Multiple reaction monitoring (MRM) transitions were optimized for these analytes (Table 2).

#### 4.2.4. Mechanistic Investigation of Berberine and Geniposide on Rhein Pharmacokinetics

(1) Assessment of berberine and geniposide on intestinal flora-mediated rhein metabolism

After 12 h fasting period, rats were administered ANIT (60 mg/kg) to induce cholestasis. Fecal was aseptically collected at 48 h post treatment and mechanically homogenized in phosphate buffer (1 g:20 mL, *w*/*v*). Following centrifugation, the clarified supernatant was harvested and designated as the fresh intestinal microbiota incubation medium.

Prior to the experiment, the bacterial solution was pre-warmed at 37 °C for 30 min. Different concentrations of berberine or geniposide were added to the incubation solution containing DH extract (0.025 g/mL crude drug), resulting in the following groups: DH (DH extract alone), DH + Berberine (L) (100 μg/mL), DH + Berberine (M) (300 μg/mL), DH + Berberine (H) (1000 μg/mL), DH + Geniposide (L) (100 μg/mL), DH + Geniposide (M) (300 μg/mL), and DH + Geniposide (H) (1000 μg/mL). The concentration ranges of berberine and geniposide (100–1000 μg/mL) used in this and subsequent in vitro transport assays were selected to reflect pharmacologically relevant estimates of their local intestinal concentrations following oral administration of the clinical equivalent dose of DHXSD. Each group was prepared in triplicate.

The anaerobic incubation was kept in a water bath (37 °C). At 0, 30, 60, and 120 min, the incubated solution (200 μL) was mixed with a 200 μL stop solution (methanol containing 10 μg/mL IS2 as the internal standard). After vortexing and centrifuging, the sample was determined by high-performance liquid chromatography (HPLC) to analyze rhein concentration. An Agilent TC-C_18_ column (4.6 × 250 mm, 5 μm particle size, Santa Clara, CA, USA) was employed for the analysis, using a constant mobile phase composition of 25% 0.1% formic acid aqueous solution and 75% methanol. The separation was carried out at a flow rate of 1 mL/min, with the column oven temperature set to 35 °C, and analyte detection performed at 254 nm.

To further investigate whether berberine’s effect on conjugated rhein metabolism involves modulation of β-glucosidase activity, we examined its influence on the enzyme-catalyzed metabolism of p-NPG, a specific substrate for β-glucosidase [31,32]. The intestinal bacterial incubation solution was pre-warmed under 37 °C, followed by the addition of berberine to solutions containing 1 mg/mL p-NPG, creating two experimental groups: p-NPG alone and p-NPG + Berberine (each in triplicate). Under anaerobic conditions at 37 °C, 0.2 mL aliquots were acquired at time points of 0, 30 min, and 1, and 2 h, mixed with 0.5 M NaOH to stop the reaction. The absorbance of the product p-NP was then quantified at 405 nm.

(2) Evaluation of rhein absorption under berberine and geniposide treatment in the everted intestinal sac model

Rhein absorption was investigated via the everted intestinal sac model in successfully modeled cholestatic rats. Following anesthesia, a 10 cm intestinal segment (starting 10 cm distal to the duodenum) was isolated through midline laparotomy, carefully dissected free of mesentery, and immediately placed in ice-cold oxygenated Tyrode’s solution (composition: NaCl 8 g/L, KCl 0.2 g/L, CaCl_2_ 0.4 g/L, KH_2_PO_4_ 0.05 g/L, NaHCO_3_ 1 g/L, MgCl_2_ 0.1 g/L, glucose 1 g/L; pH adjusted to 7.4; continuously gassed with 5% CO_2_/95% O_2_). The intestinal segment was everted, filled with 1 mL of the same oxygenated, drug-free Tyrode’s solution (serving as the serosal receiving compartment), and vertically mounted in an organ bath containing 20 mL of Tyrode’s solution with different drug additions (i.e., the mucosal side, maintained at 37 °C with 5% CO_2_/95% O_2_ oxygenation). The drugs under investigation (rhein, berberine, and geniposide) were all added to the mucosal (organ bath) side. Samples (0.2 mL) were acquired from the serosal (inner) compartment at 0, 0.5, 1, and 1.5 h, with the same volume of fresh, drug-free Tyrode’s solution replenished after each sampling. The experimental groups included: rhein alone (40 μg/mL); rhein (40 μg/mL) combined with berberine at low (100 μg/mL), medium (300 μg/mL), or high (1000 μg/mL) concentrations; and rhein (40 μg/mL) with geniposide at low (100 μg/mL), medium (300 μg/mL), or high (1000 μg/mL) concentrations, with all samples processed and analyzed using the aforementioned HPLC method. Tissue viability throughout the 1.5 h incubation was confirmed by monitoring the release of LDH into the incubation medium, a well-established indicator of cellular membrane integrity [33], consistent with common practice in ex vivo intestinal models.

(3) BCRP-MDCKII cell model for evaluating berberine and geniposide effects on rhein transport

The cytotoxicity of rhein, geniposide, and berberine (0–100 μM) was first evaluated in BCRP-MDCKII cells using the CCK-8 assay to determine non-toxic concentrations for subsequent transport experiments. Following an established method [34], cells were pre-incubated for 15 min with blank Hanks’ balanced salt solution or Hanks’ balanced salt solution containing berberine/geniposide (3–30 μM), then treated for 60 min with rhein (10 μM) alone or in combination with berberine/geniposide (3–30 μM). After rinsing with ice-cold phosphate-buffered saline, cells were disrupted via repeated freeze–thaw cycles and processed for HPLC analysis using the previously described method.

The concentration-dependent effects of berberine on BCRP-mediated rhein transport were further investigated using identical experimental procedures. Cell treatments included rhein (10 μM) combined with berberine (0, 0.3, 1, 3, 10, 30, or 100 μM), followed by the same sample processing and analytical methods as described above.

(4) Docking-based analysis of berberine’s inhibition on BCRP-mediated rhein transport

To gain insights into the inhibitory mechanisms of berberine on BCRP, molecular docking studies were performed using AutoDock Vina (version 1.2.7). The high-resolution cryo-EM structure of human BCRP (PDB ID: 8QCM) served as the receptor. The active site was defined based on the binding of internal ligands. Pre-docking preparations of the protein and ligand were done via AutoDockTools (version 1.5.6), and PyMOL (version 2.1) was used to create a grid box (center: x: 122.89, y: 129.72, z = 134.7; size: 45 × 45 × 45 Å). Docking simulations were then run with Vina’s default settings, and the binding modes along with binding free energies were recorded.

#### 4.2.5. Data Analysis

Pharmacokinetic parameters were calculated using Drug And Statistics software (version 3.0). Intergroup comparisons were performed by one-way ANOVA followed by Tukey’s post hoc test for multiple comparisons. A *p*-value < 0.05 was considered statistically significant.

## 5. Conclusions

Integrating pharmacodynamic and pharmacokinetic analyses, this study elucidates that the classical herb–herb combination in DHXSD significantly enhances the systemic exposure of anthraquinones—most notably rhein—in a cholestatic model compared to DH monotherapy. We identified and validated a primary molecular mechanism underpinning this synergy: berberine (from HB) potently inhibits the intestinal efflux transporter BCRP, thereby reducing the intestinal efflux of rhein and substantially increasing its oral bioavailability. Although geniposide (from ZZ) exhibited limited or context-dependent effects on rhein transport in our assays, its potential contributions through other synergistic pathways warrant future investigation. Collectively, this work demonstrates that targeted modulation of a key efflux transporter (BCRP) by a co-administered natural product can decisively reshape the pharmacokinetic profile of a partner drug. Beyond the specific formula, this elucidates a fundamental pharmacokinetic interaction principle within a classical herbal formula and proposes a translatable strategy for overcoming the bioavailability challenges of poorly absorbed therapeutics through rational combination. While the present work establishes a key intestinal mechanism, the full pharmacokinetic network of DHXSD, including potential hepatic components and contributions from other herbs, presents an important avenue for future research.

## Figures and Tables

**Table 1:** Pharmacokinetic parameters of anthraquinone components in cholestatic rats (mean ± SD, *n* = 6).

Compounds	Group	Cmax (ng/mL)	T_max_ (h)	AUC (ng·h/mL)	t_1/2_ (h)
Rhein	DH	793.33 ± 180.84	0.31 ± 0.16	3094.15 ± 537.50	4.11 ± 0.48
	DH+MX	560.23 ± 222.34	1.03 ± 0.86	3084.38 ± 1015.56	2.58 ± 1.21
	DHXSD	1553.97 ± 513.63 **^,##^	1.45 ± 2.55	5722.45 ± 868.32 **^,##^	4.21 ± 1.13
Aloe emodin	DH	38.95 ± 31.97	0.35 ± 0.18	110.95 ± 49.20	4.88 ± 0.97
	DH+MX	24.27 ± 8.88	0.68 ± 0.38	97.74 ± 39.07	3.73 ± 1.44
	DHXSD	73.31 ± 41.21 **^,##^	0.42 ± 0.30	184.39 ± 53.79 *^,#^	4.75 ± 1.35
Emodin	DH	54.84 ± 31.31	0.81 ± 0.70	195.41 ± 83.95	5.03 ± 1.38
	DH+MX	43.74 ± 18.98	1.50 ± 1.22	214.49 ± 100.88	7.20 ± 4.34
	DHXSD	58.44 ± 27.81	2.29 ± 2.27	276.99 ± 72.38	5.92 ± 2.81
Chrysophanol	DH	21.49 ± 10.17	0.60 ± 0.71	183.62 ± 54.24	3.44 ± 0.95
	DH+MX	19.01 ± 8.54	3.58 ± 2.69	209.52 ± 141.52	4.53 ± 1.19
	DHXSD	48.56 ± 15.46 **^,##^	2.50 ± 2.48	288.57 ± 92.85	4.36 ± 1.19
Physcion	DH	4.34 ± 1.21	4.12 ± 4.99	45.09 ± 10.54	5.27 ± 1.74
	DH+MX	6.23 ± 2.18	3.67 ± 1.51	70.27 ± 25.08	5.76 ± 1.62
	DHXSD	7.77 ± 1.77 *	2.88 ± 2.79	62.19 ± 14.68	4.22 ± 0.61

DH: Da Huang, DH+MX: Da Huang plus Mang Xiao, DHXSD: Da-Huang-Xiao-Shi Decoction. Compared to DH, * *p* < 0.05, ** *p* < 0.01. Compared to DH+MX, ^#^ *p* < 0.05, ^##^ *p* < 0.01.

**Table 2:** Quantitative determination of MRM parameters for anthraquinone components and the internal standard.

Compounds	Q1 (Da)	Q3 (Da)	DP (V)	CE (eV)	CXP (V)
Rhein	283.0	238.9	−10	−38	−18
Aloe emodin	269.0	239.8	−90.5	−33	−19
Emodin	268.9	225.1	−123	−35	−15
Chrysophanol	253.1	224.9	−75	−38	−20
Physcion	283.1	240.1	−120	−36	−20
Mycophenolic acid (IS1)	319.0	191.0	−250	−38	−20

DP: declustering potential; CE: collision energy; CXP: collision cell exit potential.

## Data Availability

The original contributions presented in this study are included in the article and Supplementary Material. Further inquiries can be directed to the corresponding authors.

## Supplementary Materials

The following supporting information can be downloaded at: https://www.mdpi.com/article/10.3390/ph19030492/s1, Table S1: Calibration curves, lower limit of quantitation (LLOQ) and the detected ions of the target compounds. Table S2: Precision and accuracy of the target compounds in quality control samples. Table S3: Contents of major compounds in DH, DH+MX, and DHXSD samples (mean ± SD, *n* = 6).

## Abbreviations

ALT	alanine aminotransferase
ANIT	α-naphthylisothiocyanate
AST	aspartate aminotransferase
AUC	area under the concentration–time curve
BCRP	breast cancer resistance protein
CE	collision energy
C_max_	maximum concentration
CXP	collision cell exit potential
DH	Da Huang
DP	declustering potential
HB	Huang Bo
HPLC	High-Performance Liquid Chromatography
IS	internal standard
LLOQ	Lower limits of quantifications
MRM	multiple reaction monitoring
MX	Mang Xiao
p-NP	p-nitrophenol
p-NPG	p-nitrophenyl-β-D-glucopyranoside
t_1/2_	half-life of elimination
TBA	total bile acids
T_max_	time to reach the maximum concentration
TCM	traditional Chinese medicine
UPLC–MS/MS	ultraperformance liquid chromatography–tandem mass spectrometry
UDCA	ursodeoxycholic acid
ZZ	Zhi Zi
