# Supplementary material for: Berberine-Mediated BCRP Inhibition Enhances Systemic Exposure of Rhein: A Study to Unravel the Pharmacokinetic Basis of Synergy in Da-Huang-Xiao-Shi Decoction
Source: Pharmaceuticals (Basel). 2026 Mar 17;19(3):492. doi: 10.3390/ph19030492 (PMC13028883; doi:10.3390/ph19030492)
Supplement: Supplementary file 1 [file pharmaceuticals-19-00492-s001.zip › pharmaceuticals-4134435-supplementary.pdf]

Supplementary Materials

The quantitative method of multiple target compounds in Da-Huang-Xiao-Shi Decoction (DHXSD) used in this study was adopted from a previously established and validated methodology [1]. The following describes its specific application to the current sample set, including instrumental conditions, sample preparation, and verification of key analytical performance parameters within the present analytical batch.

1. High-Performance Liquid Chromatography-Mass Spectrometry (HPLC-MS) Methods

Separation was achieved on an UltiMate 3000 UHPLC System (Thermo Fisher Scientific, USA) equipped with a Dikma ODS C_{18} column (4.6 × 150 mm, 5 μm) maintained at 35 °C. The mobile phase was composed of water containing water (0.1% acetic acid and 2 mM ammonium acetate) (A) and acetonitrile (B), with the following elution program: 0–1 min, 10% B; 1–7 min, 10–13% B; 7–20 min, 13–35% B; 20–21 min, 35–45% B; 21–30 min, 45–80% B; 30–34 min, 80% B; 34–34.1 min, 80–10% B; and 34–36.1 min, 10% B. The autosampler was set at 4 °C.

An LTQ-Orbitrap Elite mass spectrometer (Thermo Fisher Scientific) equipped with an electrospray ionization (ESI) source served as the platform for mass spectrometric analysis. The mass analyzer was operated in the selected ion monitoring (SIM) mode with a mass resolution of 60,000. The full scan range was set to m/z 100–1000. For quantification, the target ions were the precursor ions of each analyte, which were selected based on their optimal signal intensity and stability observed during method development. The exact masses of these precursor ions were extracted from the full scan data using a narrow mass tolerance window of ≤ 5 ppm to ensure high specificity. Other parameters were as follows: sheath gas flow, 45 arb; auxiliary gas flow, 15 arb; sweep gas flow, 0 arb; capillary temperature, 350 °C. Data acquisition and processing were performed using Xcalibur software (Thermo Fisher Scientific).

2. Assessment of Analytical Method in the Current Study

To ensure accurate quantification in the analytical batch, fresh calibration curves were prepared and analyzed concurrently with the test samples. Under the optimized chromatographic conditions, all target analytes exhibited symmetric peak shapes with no significant tailing, and the retention times in the test samples matched those of the corresponding reference standards. The reproducibility of the method was confirmed by a relative standard deviation (RSD) of less than 2% for the retention times of monitored ions in quality control (QC) samples, demonstrating excellent system stability. Quantification was performed using the linear regression equation derived from the batch-specific calibration curve. The monitored ions, regression equations, correlation coefficients (r), linear ranges, and lower limits of quantification (LLOQ) for all target analytes are listed in Table S1. All calibration curves showed excellent linearity ($r > 0.991$) over the tested concentration ranges.

Table S1 Calibration curves, lower limit of quantitation (LLOQ) and the detected ions of the target compounds

Compounds	Calibration curve	r	*m/z*	Detected ion	Linear range (μg/mL)	LLOQ (μg/mL)
rhein	Y=0.09*X+0.02	0.9956	283.0252	$[M-H]^-$	1.60-51.20	1.60
aloe-emodin	Y=0.47*X+0.05	0.994	269.046	$[M-H]^-$	0.40-12.80	0.40
emodin	Y=3.19*X+0.15	0.993	269.046	$[M-H]^-$	0.40-12.80	0.40
chrysophanol	Y=0.09*X+0.03	0.991	253.0501	$[M-H]^-$	0.80-25.60	0.80
physcion	Y=0.05*X+0.01	0.995	283.0623	$[M-H]^-$	0.40-12.80	0.40
berberine	Y=57.45*X+3.29	0.994	336.1245	M^+	0.10-3.20	0.10
phellodendrine	Y=22.16*X+5.16	0.997	342.1708	M^+	0.40-12.80	0.40
geniposide	Y=1.49*X+0.48	0.991	411.1233	$[M+Na]^+$	1.68-53.75	1.68

The precision of the method was verified by repeatedly analyzing (n=5) the quality control (QC) samples at three concentrations. The RSDs for all target analytes were below 5.00% (Table S2). The accuracy of the quantitative procedure was monitored through the recovery of the QC samples. The measured concentrations of all QC samples fell within ±15% of their nominal values, with an overall mean recovery ranging from 95.20% to 102.80% (Table S2), demonstrating satisfactory accuracy for batch analysis.

Table S2 Precision and accuracy of the target compounds in quality control samples

Compounds	Conc. (μg/mL)	Found (μg/mL)	RSD (%)	Accuracy (%)
	4.80	4.93 ± 0.18	3.73	102.80
rhein	12.80	12.31 ± 0.21	1.71	96.20
	38.40	38.86 ± 1.13	2.91	101.20
	1.20	1.17 ± 0.01	0.56	97.40
aloe-emodin	3.20	3.21 ± 0.11	3.27	100.40
	9.60	9.68 ± 0.16	1.63	100.80
	1.20	1.21 ± 0.03	2.14	101.20
emodin	3.20	3.29 ± 0.09	2.79	102.80
	9.60	9.60 ± 0.35	3.67	100.00
	2.40	2.43 ± 0.04	1.62	101.20
chrysophanol	6.40	6.50 ± 0.04	0.54	101.60
	19.20	19.01 ± 0.36	1.89	99.00
	1.20	1.16 ± 0.01	1.03	97.00
physcion	3.20	3.17 ± 0.05	1.66	99.20
	9.60	9.83 ± 0.28	2.81	102.40
	0.30	0.30 ± 0.01	4.02	100.40
berberine	0.80	0.82 ± 0.03	3.60	102.00
	2.40	2.41 ± 0.10	4.26	100.40
	1.20	1.17 ± 0.01	0.56	97.40
phellodendrine	3.20	3.26 ± 0.09	2.64	101.80
	9.60	9.52 ± 0.34	3.59	99.20
	2.40	2.36 ± 0.06	2.73	98.20
geniposide	6.40	6.09 ± 0.13	2.15	95.20
	19.20	19.16 ± 0.91	4.77	99.80

The complexity of the herbal decoction matrix necessitated an assessment of potential matrix effects caused by co-extracted components. This assessment was performed using representative herb-omitted decoctions. Specifically, to evaluate the matrix influence on the quantification of anthraquinones from Da Huang (DH), a Da Huang-omitted decoction was prepared by decocting the remaining three herbs (Mirabilitum, MX; Zhi Zi, ZZ; and Huang Bo, HB) following the standard procedure. Known concentrations of the target anthraquinone markers (rhein, emodin, aloe-emodin, chrysophanol, and physcion) were spiked into both the Da Huang-omitted decoction matrix and a pure solvent. The analyte responses (peak areas) in the two sets of samples were then compared. The results, expressed as the matrix factor (peak area in matrix / peak area in solvent), along with similar assessments performed for other analyte classes, demonstrated that the coexisting matrix did not cause significant ion suppression or enhancement. The matrix effects for the target compounds were within

an acceptable range of 85% to 115%, with RSDs less than 4.29%.

3. Sample analysis

The powdered extracts of DH, DH+MX, and DHXSD were accurately weighed. Components were extracted by ultrasonication in 10 mL of methanol for 30 min, followed by centrifugation at 16,000 × g (4 °C). A 20 μL aliquot of the supernatant was diluted 7-fold with an internal standard solution and centrifuged again (16,000 × g, 10 min). The final supernatant was analyzed by HPLC–MS for the quantification of free anthraquinones (rhein, aloe-emodin, emodin, chrysophanol, and physcion), berberine, phellodendrine, and geniposide.

For the determination of total anthraquinones (free plus conjugated forms) originating from DH, the powdered extract was processed according to the acid hydrolysis principle outlined in the *Chinese Pharmacopoeia* (2020 edition). Briefly, the extract was first refluxed with methanol for 1 h. After filtration and evaporation of the solvent, the residue was treated with 8% (v/v) hydrochloric acid (2 min of ultrasonication) and subsequently refluxed with chloroform for 1 h. The chloroform layer was separated, evaporated to dryness, and the residue was reconstituted in methanol for HPLC–MS analysis. It is acknowledged that the acid hydrolysis process may pose risks, such as the degradation of acid-labile compounds. However, as the primary objective of this study was a comparative analysis of total anthraquinone levels among the different herbal preparations (DH, DH+MX, and DHXSD), the critical factor is the consistent and uniform application of the hydrolysis protocol to all samples. This ensures that any systematic method-related bias affects all groups equally, thereby preserving the validity of the comparative conclusions regarding relative differences. The contents of free and conjugated anthraquinones (rhein, aloe-emodin, emodin, chrysophanol, and physcion), berberine, phellodendrine, and geniposide in the DH, DH+MX, and DHXSD samples were determined and are summarized in Supplementary Table S3.

Table S3 Contents of major compounds in DH, DH+MX, and DHXSD samples (mean±SD, n=6)

Compounds	Contents (μg/g crude drugs)		
	DH	DH+MX	DHXSD
free-rhein	263.2 ± 5.3*	264.8 ± 10.6*	280.6 ± 7.0

conjugated-rhein	466.0 ± 17.0*	451.6 ± 9.1*	488.4 ± 17.7
free-aloe emodin	76.4 ± 4.0*	78.7 ± 3.6*	86.4 ± 4.7
conjugated-aloe emodin	283.8 ± 13.3*	292.0 ± 6.8*	312.7 ± 14.4
free-emodin	78.6 ± 2.4*	80.1 ± 4.1*	67.1 ± 2.0
conjugated-emodin	257.9 ± 11.6	255.4 ± 11.9	293.1 ± 19.3
free-physcion	138.5 ± 5.7*	139.0 ± 8.6*	151.0 ± 4.9
conjugated-physcion	52.4 ± 1.7	51.5 ± 1.5	54.1 ± 2.6
free-chrysophanol	58.9 ± 1.5*	56.9 ± 1.2*	71.8 ± 1.8
conjugated-chrysophanol	271.1 ± 8.0	264.8 ± 11.6	260.5 ± 9.0
berberine	ND	ND	10921.4 ± 93.4
phellodendrine	ND	ND	871.3 ± 3.5
geniposide	ND	ND	5762.9 ± 82.7

ND: Not detected. $*p < 0.05$, compared to the DHXSD extract. Statistical significance for each individual compound was determined by one-way ANOVA followed by Dunnett's post-hoc test.

References:

1. Li, H.; Jin, J.; Xue, H.; Li, Y.; Wang, T.; Shi, R.; Ma, Y. Determination of multiple active constituents in Da-Huang-Xiao-Shi decoction using HPLC-LTQ-Orbitrap mass spectrometry: Application in comparing the differences in the formula and its constituent herbs. *Biomed. Chromatogr.* **2022**, *36*, e5324. https://doi.org/10.1002/bmc.5324.
